# A large National Institute for Health Research (NIHR) Biomedical Research Centre facilitates impactful cross-disciplinary and collaborative translational research publications and research collaboration networks: a bibliometric evaluation study

**DOI:** 10.1186/s12967-021-03149-x

**Published:** 2021-11-27

**Authors:** Vasiliki Kiparoglou, Laurence A. Brown, Helen McShane, Keith M. Channon, Syed Ghulam Sarwar Shah

**Affiliations:** 1 NIHR Oxford Biomedical Research Centre, John Radcliffe Hospital, Oxford University Hospitals NHS Foundation Trust, Oxford, OX3 9DU UK; 2 Nuffield Department of Primary Care Health Sciences, University of Oxford, Radcliffe Primary Care Building, Woodstock Road, Oxford, OX2 6GG UK; 3 Research Support Team, IT Services, University of Oxford, Oxford, OX1 2JD UK; 4 Nuffield Department of Clinical Neurosciences, John Radcliffe Hospital, University of Oxford, Oxford, OX3 9DU UK; 5 Nuffield Department of Medicine, The Jenner Institute, University of Oxford, Old Road Campus, Oxford, OX3 7BN UK; 6 Division of Cardiovascular Medicine, British Heart Foundation (BHF) Centre of Research Excellence, John Radcliffe Hospital, University of Oxford, Oxford, OX3 9DU UK; 7 Radcliffe Department of Medicine, Medical Sciences Division, John Radcliffe Hospital, University of Oxford, Oxford, OX3 9DU UK

**Keywords:** Research Institutions, Translational Research Organisations, Research productivity, Research outputs, Collaborative research, Author networks

## Abstract

**Background:**

The evaluation of translational health research is important for various reasons such as the research impact assessment, research funding allocation, accountability, and strategic research policy formulation. The purpose of this study was to evaluate the research productivity, strength and diversity of research collaboration networks and impact of research supported by a large biomedical research centre in the United Kingdom (UK).

**Methods:**

Bibliometric analysis of research publications by translational researchers affiliated with the National Institute for Health Research (NIHR) Oxford Biomedical Research Centre (BRC) from April 2012 to March 2017.

**Results:**

Analysis included 2377 translational research publications that were published during the second 5-year funding period of the NIHR Oxford BRC. Author details were available for 99.75% of the publications with DOIs (2359 of 2365 with DOIs), and the number of authors per publication was median 9 (mean  = 18.03, SD  = 3.63, maximum  = 2467 authors). Author lists also contained many consortia, groups, committees, and teams (n  = 165 in total), with 1238 additional contributors, where membership was reported. The BRC co-authorship i.e., research collaboration network for these publications involved 20,229 nodes (authors, of which 1606 nodes had Oxford affiliations), and approximately 4.3 million edges (authorship linkages). Articles with a valid DOIs (2365 of 2377, 99.5%) were collectively cited more than 155,000 times and the average Field Citation Ratio was median 6.75 (geometric mean  = 7.12) while the average Relative Citation Ratio was median 1.50 (geometric mean  = 1.83) for the analysed publications.

**Conclusions:**

The NIHR Oxford BRC generated substantial translational research publications and facilitated a huge collaborative network of translational researchers working in complex structures and consortia, which shows success across the whole of this BRC funding period. Further research involving continued uptake of unique persistent identifiers and the tracking of other research outputs such as clinical innovations and patents would allow a more detailed understanding of large research enterprises such as NIHR BRCs in the UK.

**Supplementary Information:**

The online version contains supplementary material available at 10.1186/s12967-021-03149-x.

## Background

Translational science requires continuous research and development (R&D) for advances in scientific understanding to lead to improvements in human health. Therefore, sustainable funding models and streams are critical to support such translational science research [1]. Evidence shows that investment in large research programmes and infrastructure and the inclusion of multi-disciplinary academic and industrial partners [2] are associated with development of new products and processes, high productivity in research outputs such as research publications and patents, and commercialisation and transfer of knowledge and technology [3, 4]. The investment in R&D also leads to a variety of payback benefits, which include knowledge, research, health, political, administrative and broader economic benefits [5, 6].

In addition, translational research provides greater opportunities for multidisciplinary research collaboration which has important implications for scientists, research networks, research partners as well as research policy and outcomes [7]. In the United Kingdom (UK), translational biomedical research involves research collaboration between universities and hospitals and these collaborations become complex because of the research collaborators’ diverse structures, procedures and work settings and more importantly due to the complex nature of patients, clinical practice and healthcare delivery [8, 9].

Evaluation of these complex translational research collaborations between universities, hospitals and industry as well as between academics and clinicians from diverse disciplines is important [10] but an arduous task. However, evaluation of translational research is critical for learning, management, accountability [11] and assessing the impact of research [12, 13]. In addition, research evaluation can inform development of strategic policy about research and science, formulation of an institutional research strategy and allocation of research funding [14]. Evaluation of research can be undertaken either prior to or after the completion of the research. The former type of research evaluation involves a review of the study protocol by a Research Ethics Committee/Institutional Review Board and approval is necessary for studies involving human participants [15] whereas the post-study evaluation involves the assessment of the outputs and impacts of the research and this type of research evaluation is helpful in assessing the performance of research projects and programmes, research centres and institutions, and individual research groups [16].

Several indicators have also been developed for evaluating different types of research benefits and impacts. For example, indicators of knowledge production include number of publications, citation rates and journal impact factors [17], whereas co-authorship and co-citation networks are indicators of research capacity building, which can be assessed by bibliometric and case studies [18]. Publications (number and their impact), collaborations (international, regional and national), multidisciplinarity and patents are considered as tier one (direct) impacts of research [19]. The number of citations and highly cited publications are indicators of research quality and these are used for evaluating research and its trends [18].

Research evaluation is a periodic exercise in some countries such as the UK where the Research Evaluation Framework (REF) [20] is an important research evaluation activity that involves assessment of publicly funded research undertaken in higher education institutions (HEI). The precursor of the REF, the Research Selectivity Exercise started in 1986 [16] and it was renamed as Research Assessment Exercise (RAE), which was undertaken in 1992, 1996, 2001 and 2008 [21, 22]. In December 2014, the RAE was replaced with the REF, which assessed the quality of research (QoR) in the national HEIs [20]. In the UK, three REF evaluations have been completed to date and the fourth REF is currently underway [23]. Although REF involves assessment of different elements, research outputs such as publications and the impact of research are the key elements [24]. Based on the findings of the REF, the UK Government funding will be allocated to the HEIs [24].

### Introduction of the study setting

The role of the National Institute for Health Research (NIHR) is to improve the health and welfare of the nation through research [25]. To this end, in 2007, the NIHR established five Biomedical Research Centres (BRCs), where NHS Foundation Trusts work in partnership with the Universities [26]. One of these first five (of 20 to date) BRCs was the NIHR Oxford BRC, a research partnership between the University of Oxford and the Oxford University Hospitals NHS Foundation Trust [26]. The NIHR Oxford BRC was originally funded through a competitively awarded grant of £57 million for 5 years from April 2007 to March 2012 [1]. In the second funding round, the BRC received £95.5 million (68% higher than the first funding award) for another 5 year period from April 2012 to March 2017 and in the third competitive funding award, the BRC was successful in getting £114 million for the period starting from April 2017 to March 2022 [1], which has been extended at least until November 2022 due to the COVID-19 pandemic.

NIHR funding can have direct and indirect routes to better healthcare as well as more general benefits to local and national economy. Measuring effectiveness is important as funding for both research and treatment are finite and there is a need to prioritise spending [27]. In addition, understanding of the barriers and gaps in the pathways translating original research to health benefits requires evaluation of research outputs and metrics, which is also important for self-assessment and correction of measurements [28]. A conventional method of evaluating translational research involves assessment of academic outputs including research publications and citations, which could be better evaluated by bibliometric methods and indicators [29], research network analysis and visualization technologies [30]. With this in mind, we set about gathering the academic research outputs of our second BRC funding cycle covering the period 2012–2017 during which the BRC comprised 14 research themes and six working groups (Additional file 1: Box S1). The research themes were bigger research groups that were established for the first 5-year funding period of the BRC while the working groups were established as newer research ‘start ups’ for the second 5-year funding period and in some cases expected to be upgraded as research themes in the next BRCs, as part of the BRC’s future strategy.

## Methods

### Study objective

The objective of this study was to evaluate translational research productivity, strength and diversity of research collaboration networks and impact of research supported by the NIHR Oxford BRC during its second 5-year period from April 2012 to March 2017.

### Outcome measures

This study included three main outcome measures i.e. research productivity measured by research publications [17, 31], research collaboration mapped through co-authorship networks [32, 33], and the quality and impact of research gauged from publication citations [34, 35].

### Data

The main data included publications that were defined as those that were reported to the NIHR as the output of the NIHR Oxford BRC between 1st April 2012 and 31st March 2017, which was the second 5-year funding period for the BRC. Individual papers were identified by staff involved in research facilitation within the BRC and from Bodleian Healthcare Libraries in the University of Oxford. Inclusion criteria supplied by NIHR for publications and stipulate amongst other things that “the work was funded/supported by the NIHR funding” [36].

### Locating digital object identifiers (DOIs) and metadata

Initially, we cleaned the publications records and then each research article was matched with its digital object identifier (DOI), which is a unique identifier that makes obtaining further information such as citation data possible. As DOIs were available for only a fraction of the publications when first recorded, a first step was to use the title field to question the Crossref API (https://api.Crossref.org). Where the original record was partial or unmatched after this process, the Crossref text query tool for matching references (https://apps.Crossref.org/simpleTextQuery) was used alongside manual searches of the bibliographic databases i.e., PubMed and EuroPMC and finally further internet searches where required. This process produced a single DOI for each of the publications in the original list (where one existed).

### Using digital object identifiers to acquire citation data

The unique identifiers for each publication (DOIs) were used to obtain current citation counts for each article from the Crossref (via the REST API, http://api.Crossref.org) and from Dimensions.ai metrics API (Dimensions is an inter-linked research information system provided by Digital Science (https://www.dimensions.ai). In a further attempt to establish a baseline for these newer metrics, Dimensions metrics API data was also collected for 500 randomly selected DOIs from Crossref.

In the citation analysis, different time periods, known as citation windows, are used such as total citations over 2 years, 5 years [37] or 10 years [38]. In the current study, we used a mix of citation window covering time periods ranging between 8 and 3 years for publications published between 2012 and 2017 respectively.

### Author numbers per publication

The total number of authors was calculated, using the author field of the Crossref citations. Many research publications also reference research consortia, often in place of individual authors where research consortia were described, and a full list of authors was apparent in the article or appendices.

### Production of co-authorship networks

All the DOIs were used to obtain lists of authors for each publication, as a whole, or equal-sized lists of DOIs representing the start, middle, and end of the funding period being studied. These lists of DOIs were used to construct a series of author co-authorship (association) networks, using Python scripts (Notebook ‘D’) (please see, Additional file 1: Figure S1). Where individual authors were associated with more than one research group, all associations were recorded, with the most prevalent used as the primary group (or type of group) for the author. Networks were also explored in the program VOSviewer [39] (version 1.6.11, https://www.vosviewer.com/) for comparison (included in analysis extra notebooks).

The resulting network files (.gml or .GEXF) were exported for visualization analysis in the Gephi (version 0.9.2, https://gephi.org/) [40]. All networks were analysed within Gephi to obtain measure of complexity (nodes and edges) and connectivity (average path length) and to filter networks for final figures. Where metrics were also calculated in the Python Networkx library the results were identical to those from Gephi.

### General methodology—data availability and tools used

The final analysis for the metrics was run on the 27th January 2021 (when data was obtained from the respective APIs). Python (Jupyter) notebooks are available describing the entire analysis from the original curated list of publications, through to the lists of DOIs used to generate the author networks. Majority of the analysis was run from these notebooks, except for a final manual check of the available titles and identifiers. The analyses make use of a number of packages from the PyData ecosystem, including Jupyter [41], IPython [42], Pandas [43, 44], Numpy [45], Scipy [46], Holoviz libraries (Bokeh [47], Hvplot, Holoviews [48], Panel, Networkx [46], Requests, FuzzyWuzzy and Habanero for the Crossref API).

We created different notebooks for the analysis (Additional file 1: Figure S1). All data are available in GitHub Repository [49] and a snapshot of code and data used (including network files) have been uploaded on the Zenodo Repository [50].

## Results

### Research publications

A total of 2377 publications were reported to the NIHR as the academic research output for the period from 1st April 2012 to 31st March 2017 (Table 1). Matching this list to Crossref records by title and DOI (or by full record), followed by a manual check of outstanding references allowed DOIs to be identified for all but 12 of these publications. Those publications still lacking DOIs after this process were commentaries, lecture notes, or book chapters without DOIs assigned. In a couple of cases DOIs were broken or unregistered (these were reported). This left 2365 of 2377 with valid DOIs (99.5%) (Table 1).

**Table 1:** Total publications, unique digital object identifiers and citations

	Count (n)	% (of DOIs)
Total publications in manually collated list	2377	100 (–)
Digital object identifiers (DOIs) found with Crossref API	2365	99.50 (100)
Data from Dimensions.ai metrics API	2364	99.45 (99.96)
Citations data in Crossref	2361	99.33 (99.83)

### Research collaboration and authorship

**Fig. 1:**
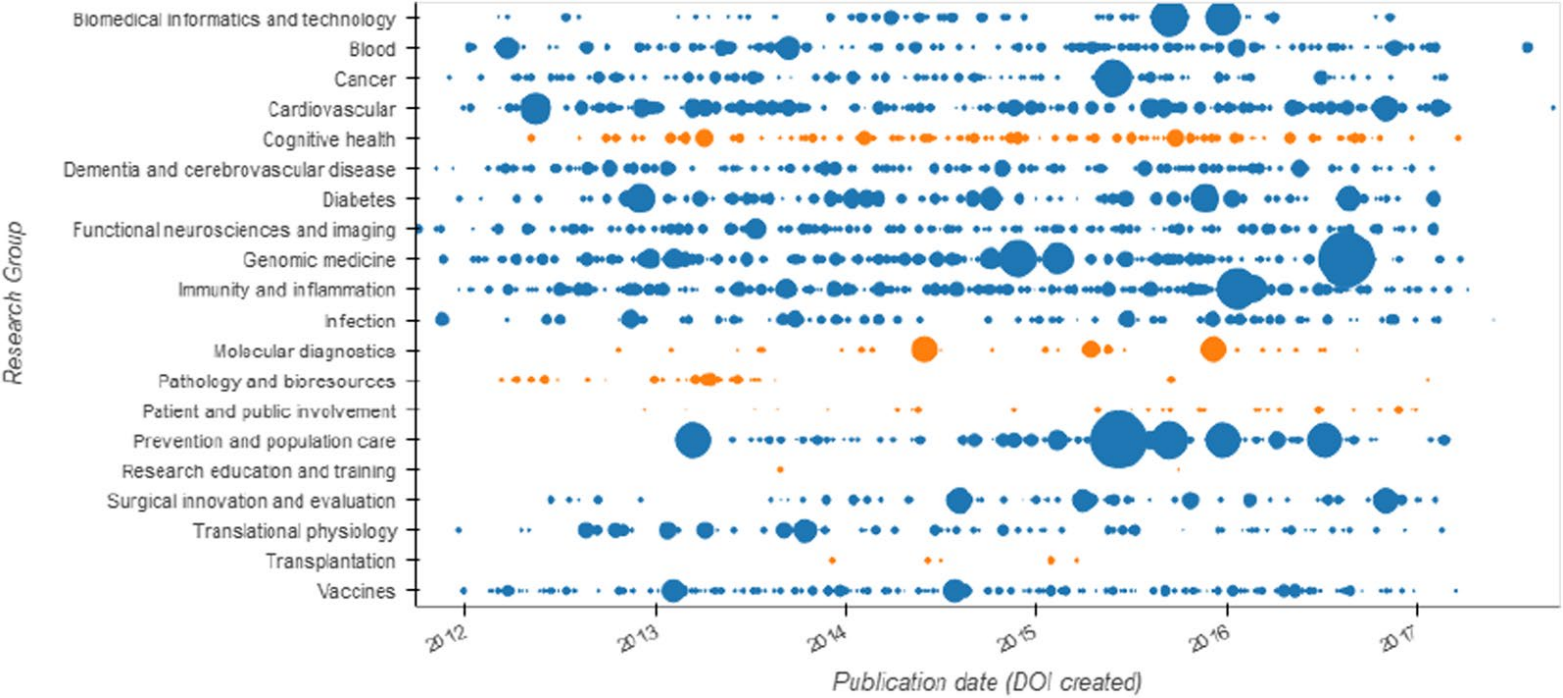
NIHR Oxford BRC publications between April 2012 and March 2017 divided by research themes (blue) and working groups (orange). Each node is a publication with a DOI, and the size of the node relates to the field citation ratio for that publication as of 27th January 2021

Two thousand one hundred and thirty-five publications were reported by all 14 Research Themes (established groups prior to this funding period) and 219 publications by six Working Groups (Fig. 1). Additionally, 23 publications were reported by ‘Other’ research groups i.e., Ethics group and Health Economic group, which were formal groups of the BRC. Less than 5% of the publications were reported by more than one research group.

**Fig. 2:**
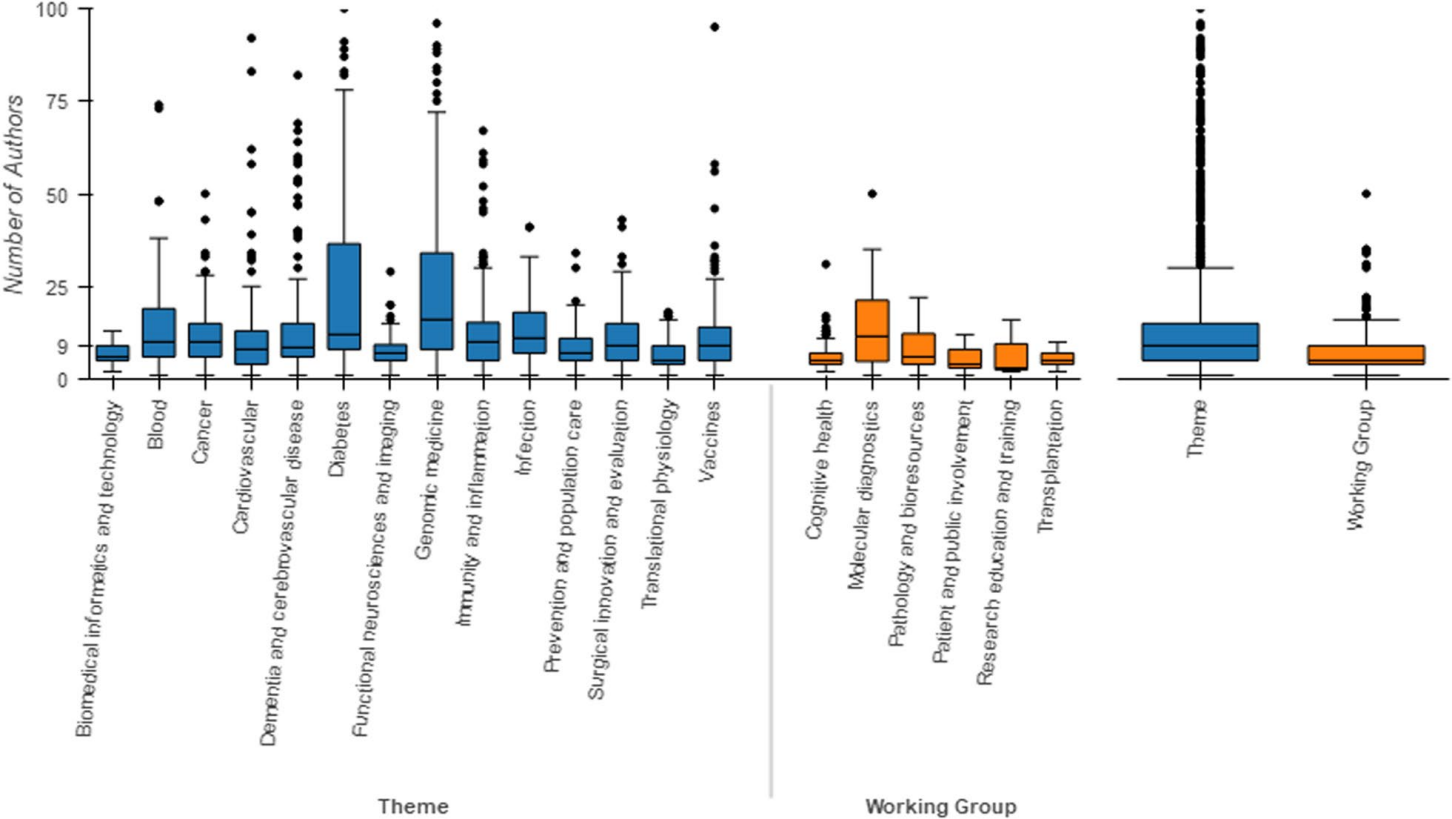
Authors per publication by research themes and working groups. Boxplot for numbers of authors (box  =  median and quartiles, whiskers  =  1.5 ×  Interquartile range)

Author details were available via Crossref for 2359 of the 2377 original listed publications (99.75% of the 2365 with DOIs). Although some publications were the work of a single author, the level of collaborative work within the NIHR Oxford BRC was notable, as were the extremes. The overall median authors number was 9, while the greatest number of authors was 2467 [51]. The mean authors number of these publications was 18.03 (SD 3.63), but it was clear that this figure was inflated by outliers, a few mega-author papers [17, 52]. In fact, 57 publications had more than 100 listed authors. The average number of authors varied greatly within and between Research Themes and Working Groups (Fig. 2). Research Themes had a median of nine authors in comparison to a median five authors for the Working Groups (Fig. 2).

### Co-authorship networks

One possible way of looking at how a large research group functions is to examine connections between researchers as a series of edges (co-authors) between nodes (individual authors). Considering the 2365 publications examined and the high average authorship, it was not surprising that the derived association network of authors was substantial. Without disambiguation beyond reducing names to initials, the co-authorship networks for the analysed publications comprised more than 20 thousand nodes (individual authors) and close to 4.3 million edges (co-authors) (Table 2). The entire co-authorship network of the BRC during the study period was a dense network having density of 0.021 (the number of edges as a proportion of the maximum possible) (Table 2).

**Table 2:** Attributes of co-authorship networks built from publications of the NIHR Oxford BRC (April 2012–March 2017)

Publication period	Measures of co-authorship networks^a^					Oxford nodes^b^	Authors per DOI		
	Nodes	Edges	Density	Average path length	Network diameter		Mean	Median	Max
Start	6,684	288,614	0.013	3.880	11	868	14.42	8	322^c^
Mid	8,697	786,972	0.021	3.539	10	1,006	18.27	8	679
End	10,913	3,534,201	0.059	3.032	10	1,068	21.67	9	2,467
All publications	20,225	4,292,252	0.021	3.076	7	1,606	18.03	9	

^a^Network measures derived from Gephi after summation of edge weights on import

^b^Oxford nodes are defined as those in which the word Oxford was found within the author’s primary affiliation

^c^Maximum without resolving all consortia and groups in publications (start section includes a known consortium of over 1200 individuals)

Dividing the total publications into 3 stages, the derived co-authorship networks from start, middle, and end of the second 5-year funding period of the BRC indicate a further strengthening of an existing network over time, with increasing density of connections overall (proportion of possible connections) and a decrease in the average path length (shortest route between any two individuals in the network (Table 2). Although there was a slight increase in the median authorship per publication, much of the substantial increase in authors in the last third of the second 5-year funding period of the BRC appeared to be due to occasional publications with very large author lists up to 2467 (Table 2).

**Fig. 3:**
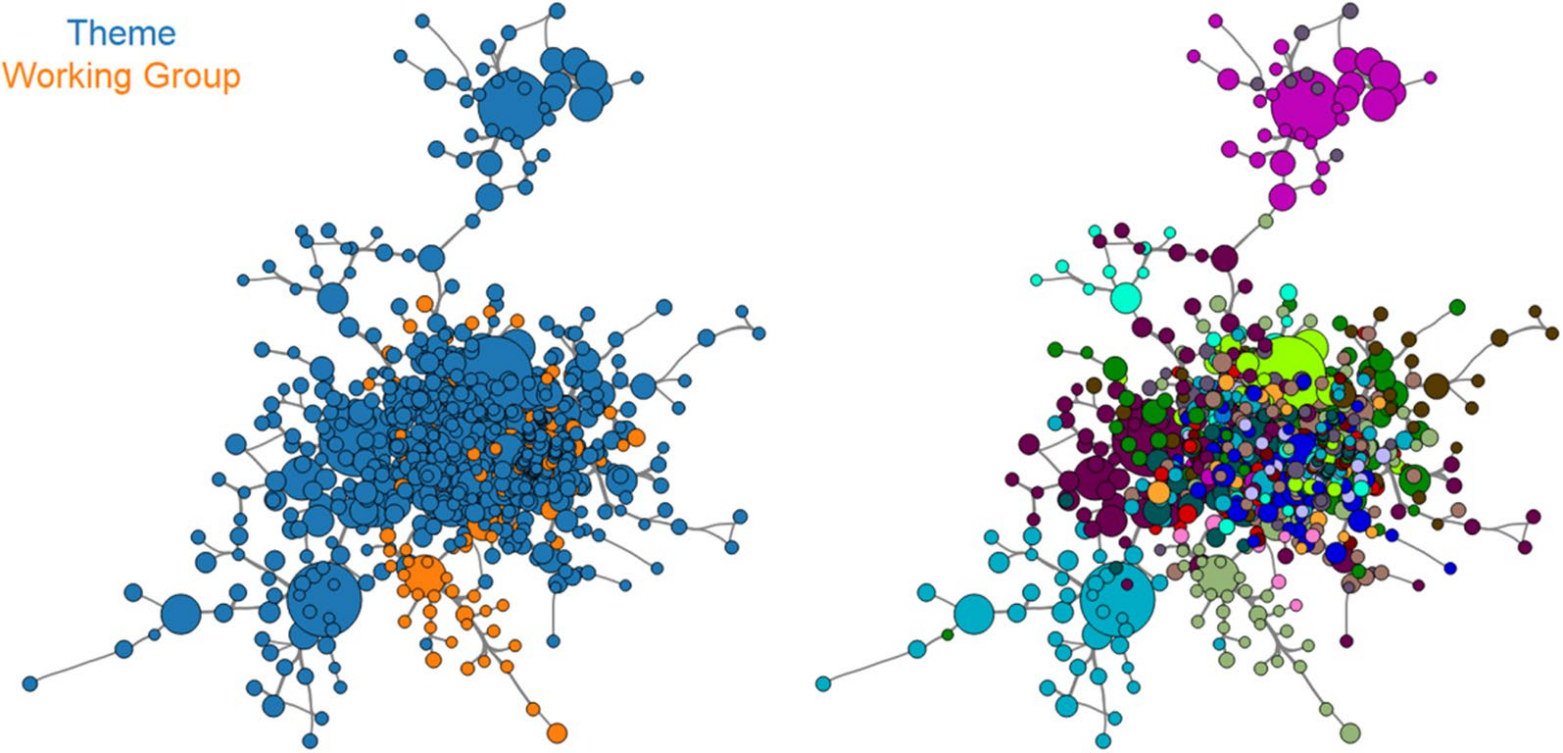
NIHR Oxford BRC authorship network showing extensive collaboration between research themes and working groups (April 2012–March 2017). **A** (Left) core of the author (relationship) network with authors primarily associated with the work from ‘research themes’ (established groups, blue), and a smaller number of authors in ‘working groups’ (orange). Each node represents an individual author (size  =  number of publications), and the edges represent the degree of co-authorship between authors (sum of weighted edges). Network diagrams are filtered (removing nodes and connections with an edge weight  < 0.5) to aid visibility. **B** (Right) core of the author network (same network as Panel **A**), but coloured by author’s most common research group, showing extensive co-authorship across research fields

Another measure of increasing collaboration with the research network is decreasing average path length over time (average number of connections needed to join any two authors). Visualizing the author network revealed tight integration with widespread interaction between research themes and working groups. The working groups, the newer research groups that were launched during the second 5-year funding period of the NIHR Oxford BRC, clearly derived from existing research networks in most cases and all were closely linked to ongoing work throughout the BRC (Fig. 3).

### Research consortia

Of the authors listed in analysed publication records (20,229 in total from Crossref), a total of 120 contained the term ‘Consortium’ with other entries also indicating multiple contributors, such as ‘Group’ [40], ‘Committee’ [6], and ‘Team’ [2]. In many cases, the size of these groupings was not stated, therefore prevented the actual authorship of the paper to be counted. It was notable that a brief examination of papers reporting as a group, there were up to 1238 additional contributors within a publication that were represented as a single ‘author’ [53].

### Publication citations and citation ratios

As of 27th January 2021, the NIHR Oxford BRC articles (published between 1 April 2012 and 31 March 2017) with DOIs were cited more than 155,000 times. The most cited article was cited over 6000 times since its publication in 2016 (Table 3).

**Table 3:** Citations and citation ratios of NIHR Oxford BRC papers published from April 2012 to March 2017

	Count^a^
Total publications with digital object identifiers (of total publications)	2365 (of 2377)
Total citations, Crossref API (n)	155, 699 (n = 2361)
Total citations, Dimensions.ai metrics API (n)	173, 995 (n = 2364)
Equivalent h-index (current n publications with at least n citations)	166; 178 (Crossref; Dimensions.ai)
Average field citation ratio from Dimensions.ai metrics API (n)	7.12 geometric mean, 6.75 median (n = 2259)
Average relative citation ratio from Dimensions.ai metrics API (n)	1.83 geometric mean, 1.50 median (n = 2300)

^a^Overall counts for NIHR Oxford BRC publications and comparison of rates of citation with similar research publications (from publication to 27th January 2021)

**Fig. 4:**
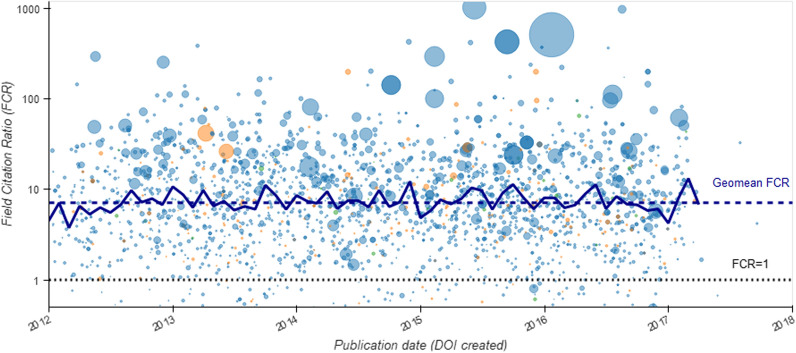
Field Citation Ratios for publications of the NIHR Oxford BRC over time coloured by research group (April 2012 – March 2017). Each node is a publication with a DOI, and the size of the node relates to the number of authors listed for the publication. Dashed blue line represents the geometric mean (g mean) FCR 7.12 for the entire publications of the NIHR Oxford BRC published during the study period and the dotted black line represents an FCR  = 1 (citation rate for a similar publication of the same age). Solid blue line is the monthly g mean of the FCR, showing a very consistent output during the funding period (1st April 2012 – 31st March 2017)

Perhaps of more value is the availability of citation ratios, where publications are compared to others of the same research field and age. The geometric mean (to avoid influence of outliers, [54]) FCR for all analysed publications was 7.12, which indicated more than 7-times the number of citations the average paper in the same research area and with same age received (Fig. 4). The publications (as assessed by citation ratios) had a similar impact across all research areas. There was a substantial variation in the sizes of Research Themes and Working Groups in different fields, but despite this the mean FCRs for all research themes and working groups were above 1 (Fig. 4).

## Discussion

In this study, we retrospectively evaluated different bibliometric measures for the NIHR Oxford BRC for its second 5-year funding period from 1st April 2012 to 31st March 2017. The studied three metrics i.e., research publications as a measure of research productivity [17, 31], co-authorship networks as a measure of research collaboration [32, 33], and publication citations as an indicator of the quality and impact of research [34, 35]. These bibliometrics are important in measuring and assessing the success of a large-scale research effort and have been used for the evaluation of research and impact in the context of biomedical research [55] including translational research [35, 56].

Our findings showed that the research productivity output of the NIHR Oxford BRC during the study period was consistently producing about 40 publications a month, every month for 5 years from April 2012 to March 2017. This linear publication rate masks differences in size and publication rates between research groups. One notable difference was the ten-fold higher rate of reported publications from the research themes, which were established research groups of the BRC prior to the study period, when compared to the working groups that were newer and established at the start of the analysed period. Regardless of research area, the citation rates on average for each research group indicate higher impact of the work compared to similar publications (all citation ratios were above 1) (Fig. 1).

Although an individual researcher level is the most common unit of analysis in studies on research productivity [57], in this study we analysed research productivity through publications at the environment level i.e., at the level of our BRC, which provides research facilities and funds as well as recognition, which are crucial factors in promoting research productivity [57] and fostering research collaboration that are positively correlated with each other [58]. Though there is a strong correlation between the quantity and impact of research such as the number of publications and number of citations respectively [34, 35], this is an area that deserves more attention [59] and maintaining a balance between the quantity and the quality of research is crucial [60]. It is also notable that evaluating research(er’s) productivity, quality and impact is not an easy task [61] and putting targets on research productivity, measured as number of publications, is a much debated and controversial issue [62].

We found a highly integrated nature of co-authorship network showing highly collaborative research working of the BRC and closer and stronger associations between researchers, which were evident from the average path length [63] and density of the network [64] as shown in Table 2. Whilst there was a perception that the BRC was important in building a robust co-authorship network, it becomes clear from this data that the network was already at least partially established at the start of second 5-year funding period of the BRC. However, there were further increases in the co-authorship network’s density and stability during the study period. It also seems that newer working groups developed with close associations to existing research themes, rather than in isolation. A true understanding of the developing research network will come from further study of the first and ongoing third 5-year funding periods of the NIHR Oxford BRC and it would be best compared to similar clinical research networks and centres, such as other NIHR BRCs in the country.

The large numbers of consortia and groups named with authorship of analysed publications is also interesting, in part because these may represent an important transition from exploratory research to an agreed vision about a route to improving healthcare and accompanying organisation and governance. Some studies have explored what factors help consortia succeed, such as scientists who are rewarded are productive and vice versa [65]. The development of a research consortium could potentially be seen as an acknowledged need for data standards and a shared voice in research and policy. The process of establishing a research consortium can be an important step in the translation of basic research to direct health benefits. However, the reporting of consortia is variable, particularly how membership relates to authorship on publications. This is another area where variations in publishing guidelines and reporting further complicates conclusion. In addition, it may be interesting to focus in the future on if there are measures (e.g., geographical diversity) that change during a large research endeavour such as the NIHR Oxford BRC.

Another important issue in relation to the performance and impact could be tackling the gender gap in scientific authorship, which could be reduced by promoting and providing fair and equitable opportunities especially to female scientists, early career researchers (both male and female) and researchers of ethnic minority background. Our earlier study on the gender equity in the authorship of scientific publications produced by researchers affiliated with the NIHR Oxford BRC revealed that although the overall proportion of female authors was lower than male authors, there were significant increasing trends of female first, last and corresponding authors and the proportions of male and female last authors were similar to their respective proportions as principal investigators in the BRC [66].

Publications analysed in this study were published in more than 700 different journals from one hundred scientific publishers with a wide variation in journal styles and formats that provide many options for how scientific progress is reported, allowing different fields and types of work to find suitable voice. However, this diversity in editorial rules and practices means substantial variability in bibliometric data. The publication process (often months of cyclical peer review and often rejection) makes it very difficult to accurately link work to a specific date. In addition, there are a multitude of minor editing decisions that can particularly affect consistency of data, from stylistic changes to lists of authors to the trimming of acknowledgements that sees funding statements removed or changed. These changes all increase the variability in the data. In recent years many publishers have come together to support efforts such as Crossref [67] to help the interchange of data within the industry improving the standards of such aggregated data.

It is also important to acknowledge to substantial changes taking place in the world of scientific publishing right now, with ‘Plan S’ an example of funders driving change to make knowledge more accessible to all [68]. Clear transparency and inclusive attempts to allow the interchange of data, as represented by efforts like Crossref, are essential and welcome.

Tracking the citation metrics of individual papers is now routine for various purposes such as checking the quality, significance and impact of research [37]. In addition, many other ways of establishing visibility and interest (or ‘impact’) being explored. However, the impact of international co-publication is amplified by self-citations, which might be unavoidable [69] but may distort or create bias in the impact of the publication or research. Hence self-citations must be corrected to gauge the actual and unbiased impact [69]. Citation metrics with more context and granularity are needed and it is useful when these are developed without restricting access. There has been progress in this area too [70]. In the hunt for better measurements of knowledge gathering and dissemination, it is essential to allow examination of the underlying data whenever possible.

There are many useful outputs from the academic research process, yet few of these are reported. This may be even truer in medical research, where valuable datasets, clinical checklists, and policy documents can often derive from research. Efforts continue to ensure the scheme for the REF measures the full value of academic study. However, the most recent REF exercise in 2014 saw 97% of the items offered up for assessment were papers or book chapters (https://hidden-ref.org/) [71]. This lack of visibility for many valuable products of research may make it even more difficult to track the steps that were essential in successful health and welfare breakthroughs.

### Research policy implications

At the heart of efforts to improve accuracy in bibliometrics is widening the availability and usage of Persistent Identifiers (PIDs). Such identifiers for publications, datasets, as well as individual researchers (ORCID [72]) and research organisations/institutions (GRID [73] and ROR [74]) will allow insights at many levels and are required to move beyond blunt journal-level measurements. This is a rapidly moving field and one where great efforts are being made to move to PIDs for many aspects of research, as much to capture the relationships between different data types as to count each one [75, 76]. Unique identifiers are thus essential for tracking anything accurately, be that a researcher, dataset, or publication. The uptake of such PIDs will be partly dependent on their visibility and availability, but also on researchers understanding how these will improve accuracy of measurement. Widespread use of PIDs, not just for publications, but also for researchers and data, must become central to publishing. Defining and adopting standard identifiers for other stages on the paths of translation to healthcare benefits [1] will also be needed.

### Strengths and limitations

We have used this study to self-assess translational research productivity and discuss current issues and the future areas of interest in the field. We have used freely available tools and data sources in this first exploration, to provide NIHR Oxford BRC members and collaborators as much data and code as possible. This process is important considering the uncertainty and limitations in some of the data as well as sources of variation in the data analysed.

## Conclusion

The substantial output of researchers supported by the NIHR Oxford BRC during its second 5-year funding period produced substantial number of research publications, which were generated by a large and complex network of translational researchers working in complex structures and consortia, which shows success across the BRC during the period of analysis. Further research involving continued improvements in and uptake of PIDs, open data and the tracking of more other research outputs such as clinical innovations and patents should give a better understanding of large research enterprises such as NIHR BRCs in the UK. In addition, variations in reporting of authorship and the lack of PIDs must be acknowledged.

## Supplementary Information

**Additional file 1: Box S1. **Research themes and working groups. **Figure S1. **Notebooks for analysis.

## Data Availability

All data are available in GitHub Repository [49], and a snapshot of code and data used (including network files) have been uploaded on the Zenodo Repository [50].

## Abbreviations

BRC: Biomedical Research Centre
DOI: Digital object identifier
FCR: Field citation ratio: a citation-based measure of scientific influence of one or more articles
FCR Mean: The average field citation ratio—indicates the relative citation performance of an article, when compared to similarly aged articles in the same fields of research category
FoR: Fields of research—a classification system covering all areas of research from Arts and Humanities to Science and Engineering
NIHR: National Institute of Health Research
PIDs: Persistent identifiers
REF: Research evaluation framework
RAE: Research assessment exercise
R&D: Research and development
RCR: Relative citation ratio
QoR: Quality of research
UK: United Kingdom
WG: Working group
