# Supplementary material for: A large National Institute for Health Research (NIHR) Biomedical Research Centre facilitates impactful cross-disciplinary and collaborative translational research publications and research collaboration networks: a bibliometric evaluation study
Source: J Transl Med. 2021 Nov 27;19:483. doi: 10.1186/s12967-021-03149-x (PMC8626935; doi:10.1186/s12967-021-03149-x)

SUPPLEMENTARY MATERIAL

**Box S.1.** Research Themes and Working Groups

**Figure S.1.** Notebooks for analysis


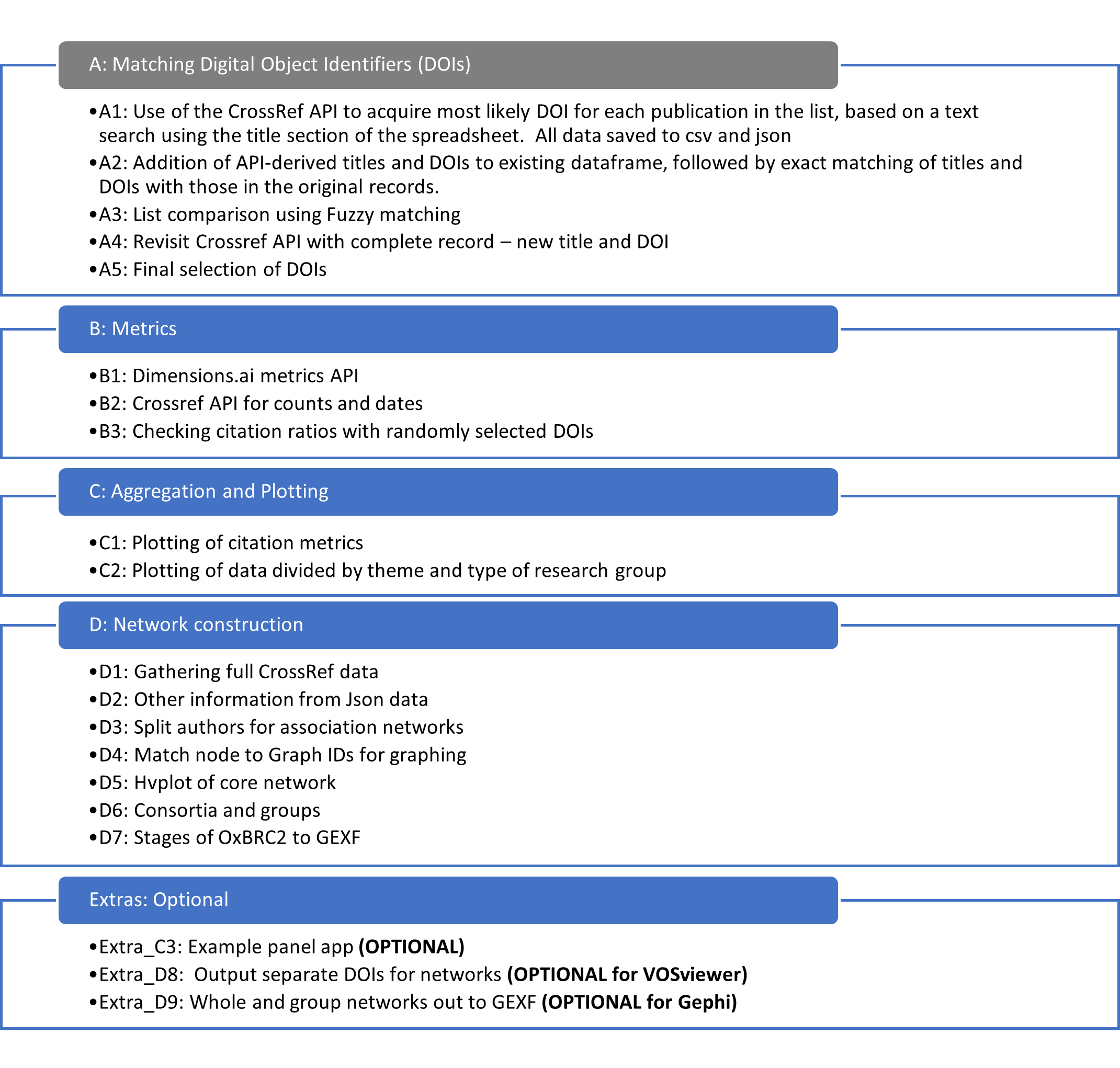

Supplement: Supplementary file 1 — Additional file 1: Box S1. Research themes and working groups. Figure S1. Notebooks for analysis. [file 12967_2021_3149_MOESM1_ESM.docx]
